# A potent tumor-selective ERK pathway inactivator with high therapeutic index

**DOI:** 10.1093/pnasnexus/pgac104

**Published:** 2022-07-01

**Authors:** Zehua Zuo, Jie Liu, Zhihao Sun, Rachel Silverstein, Meijuan Zou, Toren Finkel, Thomas H Bugge, Stephen H Leppla, Shihui Liu

**Affiliations:** Aging Institute of University of Pittsburgh and University of Pittsburgh Medical Center, Pittsburgh, PA 15219, USA; Aging Institute of University of Pittsburgh and University of Pittsburgh Medical Center, Pittsburgh, PA 15219, USA; Division of Cardiology, Department of Medicine, University of Pittsburgh School of Medicine, Pittsburgh, PA 15219, USA; Aging Institute of University of Pittsburgh and University of Pittsburgh Medical Center, Pittsburgh, PA 15219, USA; Aging Institute of University of Pittsburgh and University of Pittsburgh Medical Center, Pittsburgh, PA 15219, USA; Aging Institute of University of Pittsburgh and University of Pittsburgh Medical Center, Pittsburgh, PA 15219, USA; Aging Institute of University of Pittsburgh and University of Pittsburgh Medical Center, Pittsburgh, PA 15219, USA; Division of Cardiology, Department of Medicine, University of Pittsburgh School of Medicine, Pittsburgh, PA 15219, USA; Proteases and Tissue Remodeling Section, National Institute of Dental and Craniofacial Research, National Institutes of Health, Bethesda, MD 20892, USA; Microbial Pathogenesis Section, Laboratory of Parasitic Diseases, National Institute of Allergy and Infectious Diseases, National Institutes of Health, Bethesda, MD 20892, USA; Aging Institute of University of Pittsburgh and University of Pittsburgh Medical Center, Pittsburgh, PA 15219, USA; Division of Infectious Diseases, Department of Medicine, University of Pittsburgh School of Medicine, Pittsburgh, PA 15219, USA

**Keywords:** anthrax lethal toxin, CMG2, ERK signaling, intermolecular complementation, tumor targeting

## Abstract

FDA-approved BRAF and MEK small molecule inhibitors have demonstrated some level of efficacy in patients with metastatic melanomas. However, these “targeted” therapeutics have a very low therapeutic index, since these agents affect normal cells, causing undesirable, even fatal, side effects. To address these significant drawbacks, here, we have reengineered the anthrax toxin-based protein delivery system to develop a potent, tumor-selective MEK inactivator. This toxin-based MEK inactivator exhibits potent activity against a wide range of solid tumors, with the highest activity seen when directed toward tumors containing the BRAF^V600E^ mutation. We demonstrate that this reengineered MEK inactivator also exhibits an extremely high therapeutic index (>15), due to its in vitro and in vivo activity being strictly dependent on the expression of multiple tumor-associated factors including tumor-associated proteases matrix metalloproteinase, urokinase plasminogen activator, and anthrax toxin receptor capillary morphogenesis protein-2. Furthermore, we have improved the specificity of this MEK inactivator, restricting its enzymatic activity to only target the ERK pathway, thereby greatly diminishing off-target toxicity. Together, these data suggest that engineered bacterial toxins can be modified to have significant in vitro and in vivo therapeutic effects with high therapeutic index.

Significance Statement Many naturally occurring bacterial protein toxins have evolved to target cellular signaling pathways that are often dysregulated to drive progression of human cancers. As such, bacterial toxins serve as a potential valuable source for novel antitumor agents. Here, through reengineering the unique structural/functional domains of anthrax toxin, we describe the successful development of a potent, highly tumor-selective MEK inactivator, which is effective against a wide variety of tumors with an extremely high therapeutic index, thereby demonstrating clear advantages in both potency and specificity over the current FDA-approved small-molecule inhibitors. Importantly, we demonstrate that the immunogenicity of the toxin can be safely and efficiently overcome by a B-cell depleting regimen. These observations suggest that bacterial toxins can be re-engineered/repurposed to target human tumors.

## Introduction

Oncogenic activation of pathways that regulate cell proliferation and survival are frequently involved in human tumorigenesis. Specific mutations in the RAS–RAF–MEK–ERK pathway are associated with 46% of all human cancers, with KRAS mutations in 9% and BRAF mutations in 7% of all human cancers (1). KRAS alterations are most common in pancreatic carcinoma (72%), colorectal cancer (69%), and lung carcinoma (33%). BRAF mutations (most often V600E) are most frequent in melanoma (51%) and thyroid carcinoma (62%). Subsequently, small-molecule inhibitors of BRAF (such as Vemurafenib and Dabrafenib) and MEK (Trametinib and Cobimetinib) (2) have been developed and approved by the FDA for treatment of patients with BRAF mutations. Although small-molecule inhibitors of BRAF and MEK have some efficacy in patients with metastatic diseases, their utility is limited because they also target normal cells, causing undesirable, even fatal, side effects (3, 4). Therefore, there are critical unmet medical needs to develop more specific therapeutics that target these pathways. In this regard, many bacterial pathogens have evolved potent protein toxins to disrupt specific pathways involved in microbial pathogenesis, which are also essential for tumor development (5–8). Fortunately, these potent, naturally occurring toxins can be structurally modified to achieve high tumor specificity. One such example is anthrax lethal toxin, which targets MEK, and has the potential to be repurposed and re-engineered into a potent anticancer drug (9, 10).

Anthrax lethal toxin, secreted by *Bacillus anthracis* (the causative agent of anthrax), is a typical A–B type toxin consisting of two polypeptides: a cellular receptor-binding and delivering component termed protective antigen (PA), and an enzymatic moiety denoted as lethal factor (LF) (8, 11, 12). To gain entry into mammalian cells, PA binds to the cell surface receptors CMG2 (capillary morphogenesis protein-2) or TEM8 (tumor endothelium marker-8) (8, 11). This binding results in a proteolytic activation of PA on the cell surface by the protease furin, yielding the active PA oligomer. The PA oligomer then binds and translocates LF into the cytosol of target cells to exert its cytotoxic effects (8, 11).

The unique requirement for PA proteolytic activation on the target cell surface provides a way to re-engineer PA to be activated by a tumor-associated protease rather than furin. Therefore, we have previously successfully generated PA variants, namely PA-L1 and PA-U2, that are activated by tumor-associated proteases MMPs (matrix metalloproteinases) and uPA (urokinase plasminogen activator), respectively (Table S1, Supplementary Material) (13–17). Upon proteolytic activation on the surface of a target cell, PA oligomerizes and gains the capacity to bind LF or LF fusions. Each LF-binding site is formed by subsites from two adjacent PA protomers (18–20). Based on this fact, we have successfully generated PA variants (PA-L1-I207R and PA-U2-R200A) that depend on their intermolecular complementation to form the active LF-binding sites (20). Because these two PA variants (together termed Intermolecular Complementation version 2, or IC2-PA; Table S1, Supplementary Material) require MMPs and uPA, respectively, for activation, the action of this intermolecular complementation system relies on the presence of two distinct tumor-associated proteases, thereby achieving high tumor specificity (16, 17), and minimizing the side effects that have limited previous therapy (21).

**Figure 1.**
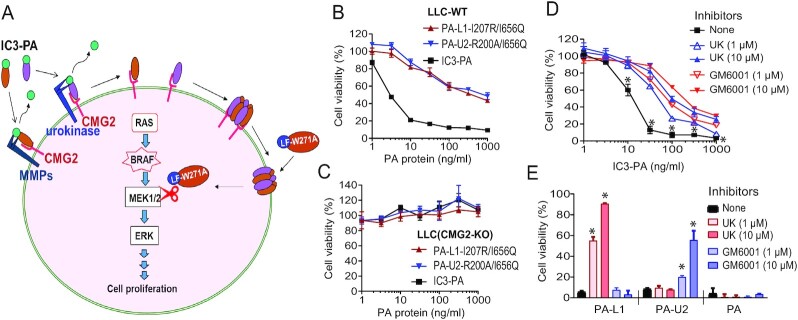
IC3-PA requires simultaneous presence of the three tumor markers MMPs, urokinase, and CMG2 for its cytotoxic action. (A) Mode of action of the reengineered anthrax toxin-based MEK inactivator. IC3-PA consists of PA-L1-I207R/I656Q and PA-U2-R200A/I656Q, which are activated by MMPs and uPA, respectively. Upon proteolytic activation, PA-L1-I207R/I656Q and PA-U2-R200A/I656Q form oligomers and gain the capacity to bind LF or LF variants. Each active LF-binding site is formed by two subsites (I207 and R200) on two adjacent hetero PA protomers PA-L1-I207R/I656Q and PA-U2-R200A/I656Q, each proving one subsite, i.e. R200 and I207, respectively. High tumor specificity of IC3-PA is derived by its selectively binding to CMG2 receptor and relying on concurrent presence of the two distinct tumor-associated proteases (MMPs and uPA) for activation. Thus, LF-derived effector proteins (such as LF-W271A) can be selectively delivered into tumor cells to target the ERK pathway. (B) IC3-PA requires intermolecular complementation of PA-L1-I207R/I656Q and PA-U2-R200A/I656Q for its cytotoxic action. Lewis lung carcinoma (LLC) cells were incubated with various concentrations of PA proteins in the presence of FP59 (100 ng/mL) for 48 h, followed by an MTT assay for assessing cell viability. IC3-PA (1 μg) = PA-L1-I207R/I656Q (0.5 μg) + PA-U2-R200A/I656Q (0.5 μg). Means ± SD. (C) IC3-PA needs binding to CMG2 receptor for its cytotoxic action. While LLC–WT cells were sensitive to IC3-PA/FP59 (in B), LLC(CMG2-KO) cells were completely resistant to the toxin. (D) and (E) IC3-PA relies on protease activities of MMPs and uPA for its cytotoxic action. LLC–WT cells were incubated with/without 1 or 10 μM GM6001 or UK371804HCL and various concentrations of PA proteins in the presence of FP59 (100 ng/mL) for 2 h. Then the cells were replaced with fresh medium without the toxin and protease inhibitors and cultured for 48 h, followed by an MMT assay for assessing cell viability (D). We also included PA-U2 and PA-L1 as additional controls, verifying that GM6001 could only block PA-L1’s cytotoxicity, and UK371804HCL could only inhibit PA-U2’s activity (E). Of note, neither inhibitor affected WT-PA's cytotoxicity. In (E), PA variants (10 ng/mL) plus FP59 (100 ng/mL) were used as in (D) in the presence of the protease inhibitors as indicated. Means ± SD.

Recently, we found that CMG2 is the major PA receptor on cancer cells and tumor stromal cells (16). Therefore, PA variants that only bind CMG2 but not TEM8 would retain antitumor activity while sparing toxicity to other tissues that express TEM8 (such as the kidneys and brain) (22, 23). In this work, we further improved IC2-PA's tumor specificity by making it specific to the major toxin receptor CMG2, which is highly expressed on both cancer cells and tumor stromal cells (16). We expected that this improved PA variant, termed IC3-PA (Table S1, Supplementary Material), combined with our novel MEK1/2-specific LF variant (see below), would form a highly tumor-selective MEK inactivator for tumor targeting (Figure 1A).

The native effector LF is a zinc-dependent metalloproteinase that cleaves and inactivates multiple mitogen-activated protein kinase kinases (MEKs), resulting in the inactivation of three key mitogen-activated protein kinase (MAPK) pathways (24, 25): the ERK (through cleavage of MEK1/2), p38 (through MEK3/6), and Jun N-terminus kinase (JNK) (through MEK4/7) pathways (8, 11). We have previously shown that LF has a potent antitumor activity when combined with the earlier versions of our tumor-selective PA delivery proteins (16, 26). Here, we hypothesized that LF's antitumor activity may largely reside in its MEK1/2 inactivation, while inactivating the p38 and JNK pathways may not increase antitumor efficacy and may largely be responsible for toxicity. In this study, we have generated and characterized a MEK1/2-specific LF variant, which displays potent antitumor activity with an extremely high therapeutic index when combined with our reengineered IC3-PA. We showed that our highly tumor-selective MEK inactivator, i.e. LF-W271A/IC3-PA, has a wide range of antitumor activity, with particular efficacy in targeting tumors harboring the BRAF^V600E^ mutation.

## Results

### Reengineered anthrax protein delivery system as a platform for highly specific tumor targeting

CMG2 is the major PA receptor on cancer cells and tumor stromal cells (16). Therefore, we reasoned that PA variants that exclusively bind CMG2 would retain their antitumor activity while largely sparing toxicity to healthy tissues that express the TEM8 receptor (such as kidneys and brain) (22, 23). Recently, kidney and spleen were found to express TEM8 as their major toxin receptor (22). PA domain-4 is responsible for binding to its receptors CMG2 and TEM8. In a structure/function study, we have identified PA domain-4 variants, i.e. PA-I656Q, PA-Y681A, and PA-L687A, that selectively use CMG2 rather than TEM8 for cellular entry. To verify this, we noted that these CMG2-selective PA variants efficiently killed CMG2-expressing Chinese hamster ovary (CHO) cells but not TEM8-expressing CHO cells when FP59 was used as an effector protein (Figure S1A, Supplementary Material). FP59 is a fusion of LFn (N-terminal PA binding domain of LF) and the catalytic domain of *Pseudomonas aeruginosa* exotoxin A that kills cells by shutting down protein synthesis through ADP-ribosylation of eEF2, after PA-mediated delivery into the cytosol (27). We chose PA-I656Q for further analyses because of its potency and high stability and yield in protein production from our lipopolysaccharides-free BA expression system (see Methods). Schild plot analyses demonstrated that PA-I656Q had markedly reduced ability to bind TEM8, but retained a comparable affinity for the CMG2 receptor when compared to wildtype (WT) PA (Figure S1B and 1C, Supplementary Material). These results validated the selectivity of PA-I656Q for the CMG2 receptor. Based on these observations, we introduced the I656Q mutation into our earlier version of the Intermolecular Complementation PA variant, termed IC2-PA (composed of PA-L1-I207R and PA-U2-R200A), yielding IC3-PA (consisting of PA-L1-I207R/I656Q and PA-U2-R200A/I656Q) that requires concurrent presence of MMPs and uPA for activation, and CMG2 receptor for cellular binding (Table S1, Supplementary Material; Figure 1A).

As expected, IC3-PA could efficiently kill mouse Lewis lung carcinoma (LLC) cells in the presence of FP59, but not the CMG2-knockout (KO) LLC cells (Figure 1B and C). Further, the cytotoxicity of IC3-PA to WT LLC cells was greatly reduced in the presence of either the MMP inhibitor GM6001 or the uPA inhibitor UK371804HCL (Figure 1D and E). Hence, IC3-PA achieves high tumor-specificity by simultaneously targeting three distinct cancer markers, i.e. tumor-associated proteases MMPs and uPA, as well as the CMG2 receptor found on cancer cells (Figure 1). As expected, with the potential improvement in safety feature IC3-PA retained the full antitumor activity of its earlier version IC2-PA (when combined with LF) in treating B16F10 melanomas grown in immunocompetent mice (Figure S2A, Supplementary Material). Interestingly, while the mice tolerated four doses of LF/IC3-PA (6.7 µg/20 µg) treatment, two out nine mice succumbed the same doses of LF/IC2-PA treatment (Figure S2, Supplementary Material).

### ERK pathway-specific LF variant as an effective antitumor effector protein

**Figure 2.**
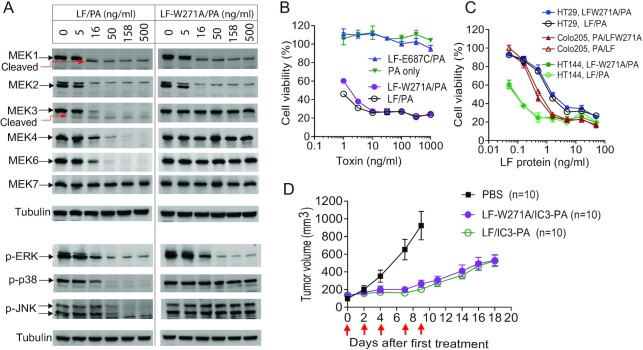
Selective inhibitory activity of LF-W271A to the ERK but not the p38 and JNK pathways. (A) MEK1/2-selectivity of LF-W271A. LLC cells were incubated with various concentrations of LF/PA or LF-W271A/PA for 3 h, followed by western blotting using anti-MEK1, -MEK2, -MEK3, -MEK4, -MEK6, and -MEK7 antibodies for assessing proteolytic cleavage of the MEKs, or using antiphospho-ERK (T202/Y204), -phospho-p38 (T100/Y182), and -phospho-JNK (T183/Y185) antibodies to evaluate activation status of these pathways. Compared to WT LT, LT-W271A had similar proteolytic activity toward MEK1/2, but lost activity to other MEKs. Consistently, by restricting inhibitory activity to the ERK pathway, LT-W271A could not disrupt the p38 and JNK pathways. Of note, MEK7 was not a target of LT. (B) LF-W271A/PA and LF/PA are equally cytotoxic to LLC cells. LLC cells were incubated with various concentrations of LF-W271A/PA or LF/PA for 72 h, followed by an MTT assay to measure cell viability. PA only and PA plus LF-E687C (LF catalytically inactive variant) were used as additional controls. Means ± SD. (C) HT144, Colo205, and HT29 cells that are dependent on the ERK pathway for proliferation are equally susceptible to LF-W271A/PA and LF/PA. The cells were incubated with various concentrations of LF-W271A or LF in the presence of 500 ng/mL PA for 72 h, followed by an MTT assay to measure cell viability. Means ± SD. (D) LF-W271A/IC3-PA and LF/IC3-PA exhibit comparable antitumor activity. LLC tumor-bearing C57BL/6 J mice were treated intraperitoneally (IP) with LF-W271A/IC3-PA (6.7 µg/20 µg) or LF/IC3-PA (6.7 µg/20 µg) as indicated by the arrows. Means ± SE.

We have shown previously that the native effector LF exhibits potent antitumor activity when combined with our earlier versions of tumor-selective PA proteins (Table S1 and Figure S2, Supplementary Material) (16, 20). However, it remains unclear whether LF's antitumor activity is due solely to inactivation of the ERK pathway, or whether the inhibitory effects on the p38 and JNK pathways are also important. To address this question and to develop potent and therapeutic tumor-selective MEK inactivators, it would be useful to generate LF variants that only inactivate MEK1/2. LF contains four functional domains: The N-terminal domain-I mediates the binding to PA oligomers; the C-terminal domain-IV catalyzes the cleavage of the substrates, and the central domain-II and –III are believed to recognize various substrates (28). Based on the LF's structure, previous mutagenesis studies, and computer-aided molecular design (28–31), a surface hydrophobic area containing residue W271 (located in domain II) was recently identified as a region that determines LF substrate specificity while minimally affecting its enzymatic activity (29). When mutating the W271 to the amino acids Ala, Val, Gly, or Arg, we found that these LF variants retained their proteolytic activity to MEK1/2, but lost activity to other MEKs (Figure S3, Supplementary Material). We chose LF-W271A for further characterization because of its stability and high yield in expression and purification. Just like WT LF/PA, LF-W217A/PA could efficiently enter cells and cleave MEK1 and MEK2. However, this variant lost its proteolytic activity toward other MEKs (Figure 2A). Consistent with these observations, while WT LF disturbed all three MAPK pathways by diminishing phosphorylation of ERK (at T202/Y204), p38 (at T100/Y182), and JNK (at T183/Y185), LF-W271A only affected the ERK pathway (Figure 2A). Interestingly, we found that MEK7 is not a substrate of either LF or LF-W271A (Figure 2A).

Next, we sought to compare the cytotoxicity of LF-W217A/PA and WT LF/PA on cancer cells, particularly those that rely on the ERK pathway for proliferation. We treated LLC cells, as well as the human cancer cell lines HT29, HT144, and A2058 cells with either LF-W217A/PA or LF/PA (Figures 2B and C). We found that LF/PA and LF-W271A/PA had equivalent cytotoxicity on these ERK-dependent cells, whereas PA only or LF-E687C/PA (catalytically inactive LT (32)) were not toxic to these cells (Figure 2B). These results demonstrate that LF-W271A/LF and LF/PA have equivalent inhibitory activity for the ERK pathway.

To examine whether LF-W217A has the same in vivo antitumor activity as WT LF, we employed a mouse LLC syngeneic tumor model. In immunocompetent LLC tumor-bearing mice, we systemically administered (via an intraperitoneal route, IP) six doses of LF/IC3-PA (6.7 µg/20 µg) or LF-W271A/IC3-PA (6.7 µg/20 µg), as indicated by red arrows in Figure 2(D). We noted that both toxins have equal antitumor activity (Figure 2D). Therefore, the ERK pathway is the dominant MAPK pathway regulating tumor growth, whereas targeting the p38 and JNK pathways appears to contribute little in this experimental setting.

### Anthrax toxin-based tumor-selective MEK inactivator shows a very high therapeutic index

**Figure 3.**
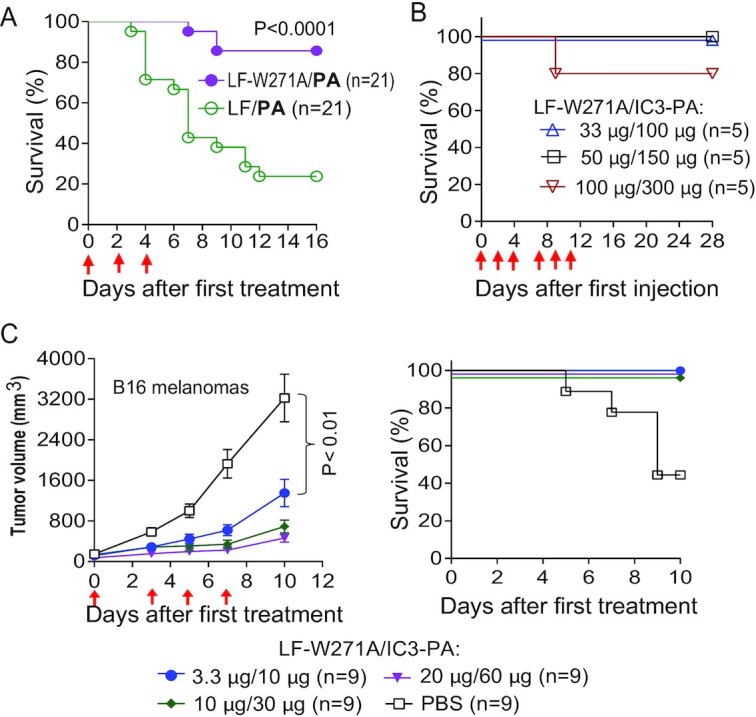
Extremely high therapeutic index of the tumor-selective MEK inactivator LF-W271A/IC3-PA. (A) Reduced in vivo toxicity of LF-W271A compared to WT LF. C57BL/6 J mice were injected (IP) with three doses of LF-W271A/PA (20 µg/20 µg) or LF/PA (20 µg/20 µg) as indicated (red arrows), with survival monitored for 2 weeks. (B) Dose-escalation studies to measure maximum tolerated doses of LF-W271A/IC3-PA. C57BL/6 J mice were injected (IP) with six doses of LF-W271A/IC3-PA as indicated (red arrows). Of note, mice tolerated six doses of 50 µg/150 µg LF-W271A/IC3-PA without any signs of malaise. Only one mouse succumbed to 100 µg/300 µg LF-W271A/IC3-PA. (C) Antitumor activity of LF-W271A/IC3-PA in syngeneic B16F10 melanomas. B16F10 melanoma-bearing mice were injected IP with various doses of LF-W271A/IC3-PA as indicated (red arrows). Survival of each treatment group was monitored (right panel). Of note, significant antitumor activity was observed for doses as low as 3.3 µg/10 µg of LF-W271A/IC3-PA. Thus, the therapeutic index of LF-W271A/IC3-PA is ≥ 15 (50 µg/150 µg ÷ 3.3 µg/10 µg) in B16F10 syngeneic tumors. Means ± SE.

While exhibiting potent antitumor activity, we reasoned that LF-W271A may have reduced in vivo toxicity since it lacks the capacity to inactivate the p38 and JNK pathways, the two major stress-activated pathways that hosts activate in order to adapt to a myriad of unfavorable conditions for survival (33–35). To test this, we challenged healthy, nontumor bearing C57BL/6 J mice with three doses of LF/PA (WT PA; 20 µg/20 µg) or LF-W271A/PA (WT PA; 20 µg/20 µg). Remarkably, while 80% of the mice given LF/PA died, only 3 out of 21 (14%) mice challenged with LF-W271A/PA succumbed (Figure 3A). This improved safety feature of LF-W271A indicated that LF-W271A/IC3-PA would likely have a high therapeutic index. To estimate the therapeutic index (maximum tolerated doses/minimum effective antitumor doses) of our tumor-targeting toxin, we performed dose-escalation studies using LF-W271A/IC3-PA. We found that mice could tolerate six doses of 50 µg/150 µg LF-W271A/IC3-PA without any obvious signs of distress (Figure 3B). We then evaluated the antitumor activity of various doses of LF-W271A/IC3-PA in immunocompetent C57BL/6 mice bearing B16F10 melanomas. Antitumor activity was observed even when LF-W271A/IC3-PA was decreased to 3.3 µg/10 µg and four injections (Figure 3C). Importantly, no deaths were observed in any of the toxin treatment groups.

Based on the fact that 3.3 µg/10 µg of LF-W271A/IC3-PA displayed significant antitumor activity to B16F10 syngeneic tumors, the therapeutic index of LF-W271A/IC3-PA was estimated at 15 (50/150 ÷ 3.3/10), an index significantly higher than the clinically available small-molecule MEK inhibitors, which have a therapeutic index close to 1 (3, 4, 36). Therefore, this supports the conclusion that LF-W271A/IC3-PA represents an anthrax toxin-based, highly tumor-selective MEK inactivator.

### Cancer cells with a BRAF^V600E^ mutation are highly sensitive to the reengineered toxin

**Figure 4.**
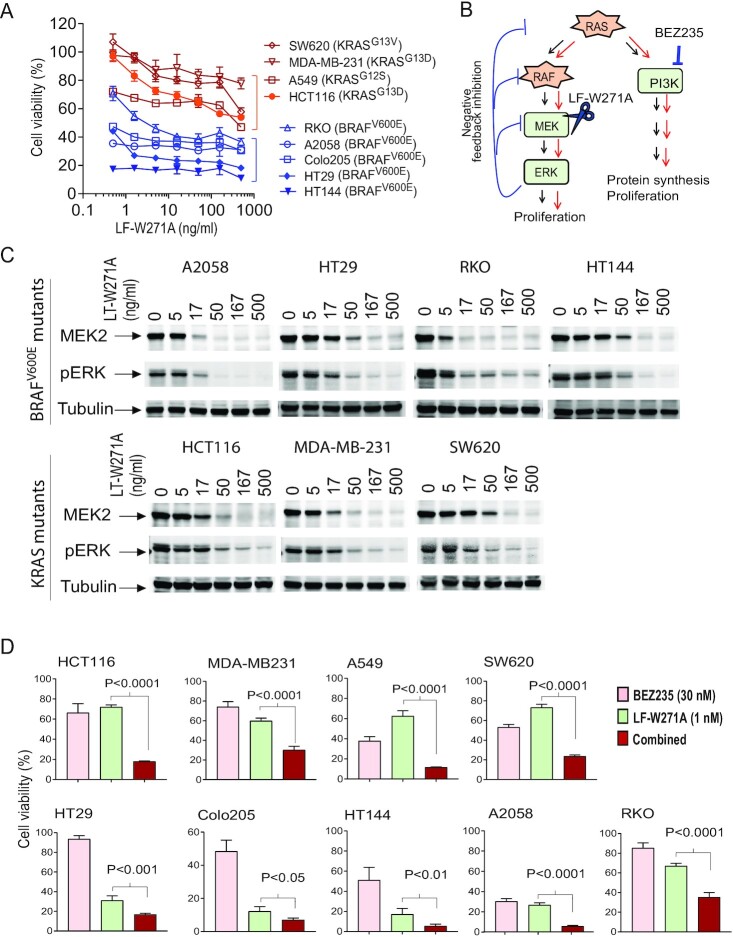
Succeptibility of human cancer cells with oncogenic BRAF or KRAS mutation to the MEK inhibition by LF-W271A. (A) Cancer cells with the BRAF^V600E^ mutation but not the oncogenic RAS mutations are more sensitive to the MEK inhibition by LF-W271A/PA. Cancer cells with the indicated mutations were incubated with various concentrations of LF-W271A in the presence of 500 ng/mL PA for 72 h, followed by an MTT assay for assessing cell viability. Means ± SD. (B) Signal transduction pathways driven by the oncogenic RAS and BRAF. Oncogenic RAS proteins can drive both the MEK–ERK and PI3K effector pathways for cell proliferation. Therefore, under the MEK–ERK inhibition, the cells with oncogenic RAS mutations may still survive via the PI3K pathway signaling. (C) Cells were incubated with various concentrations of LF/PA or LF-W271A/PA for 3 h, followed by western blotting using anti-MEK2 or anti-Phospho-ERK (T202/Y204) antibody to evaluate activation status of ERK pathway. (D) Synergistic or additive cytotoxic effect of LF-W271A/PA and BEZ235 to a set of human cancer cells. Cells were treated with LF-W271A/PA/(1 nM = 85 ng/mL each of LF-W271A and PA), BEZ235 (30 nM), or their combination for 48 h, followed by an MTT assay to assess cell viability. Unpaired two-tailed Student's t test.

Oncogenic KRAS mutations (on residue Gly^12^ or Gly^13^) and BRAF^V600E^ mutation are common in human cancers. Thus, we next characterized the susceptibility of a set of human cancer cells (Table S2, Supplementary Material) with either oncogenic KRAS mutations or a BRAF^V600E^ mutation to MEK1/2 inactivation mediated by LF-W271A. Interestingly, all the cancer cells with the BRAF^V600E^ mutation, including RKO, HT29, Colo205 human colon cancer cells, and HT144, A2058 human melanoma cells, were sensitive to LF-W271A/PA (Figure 4A; Figure S4A, Supplementary Material). In contrast, human cancer cells with oncogenic KRAS mutations (HCT116 and SW620 colon cancer cells, A549 lung and MDA-MD-231 breast cancer cells) were much less sensitive to this MEK inhibition (Figure 4A; Figure S4A, Supplementary Material). This could be explained by the notion that cells with the BRAF^V600E^ mutation are more “addicted” to MEK–ERK signaling than the cells with oncogenic RAS mutations for survival (37). To rule out the possibility that the difference in sensitivity to the toxin was due to the variance in the toxin's delivery into the cytosol, we performed cytotoxicity assay using FP59 as the effector protein, which kills cells independently on oncogenic mutations. Contrastingly, the sensitivity to FP59/PA could not be separated by BRAF vs. KRAS mutations (Figure S4B, Supplementary Material). Furthermore, the LT-W271A-induced MEK cleavage and ERK signaling disruption could occur in a dose-dependent manner in both cells with the BRAF^V600E^ mutation as well as with the KRAS mutations (Figure 4C). Because LF-W271A and LF can irreversibly cleave MEK1/2, we reasoned that their action in cytosol may last for a long period of time. To test this, we treated HT29 cells with the toxins for 2 h, then continued to culture the cells without the toxins. We found that the ERK signaling in these cells remained at disrupted status even 48 h after removal of LF-W271A/PA or LF/PA (Figure S4C, Supplementary Material).

Therefore, LF-W271A/PA appears to preferentially target cancer cells relying on oncogenic BRAF–MEK–ERK signaling for survival. The decreased sensitivity to MEK inhibition in cancer cells containing oncogenic KRAS mutations is likely due to another RAS-effector pathways (e.g. PI3K pathway) that can maintain cell proliferation and survival (Figure 4B). Supporting this, we demonstrated that the specific PI3K inhibitor BEZ235 provided synergistic or additive cytotoxic effect with LF-W271A/PA, an effect that was particularly evident in cells bearing oncogenic KRAS mutations (Figure 4C). Interestingly, although cancer cells with the BRAF^V600E^ mutation are addictive to the MEK–ERK signaling for survival, BEZ235 could further offer additive or synergistic effects with the toxin, suggesting that PI3K pathways may also contribute to survival of these cells. Taken together, we expected that our tumor-selective MEK inactivator LF-W271A/IC3-PA would be effective in treating tumors relying on the ERK pathway for survival.

**Figure 5.**
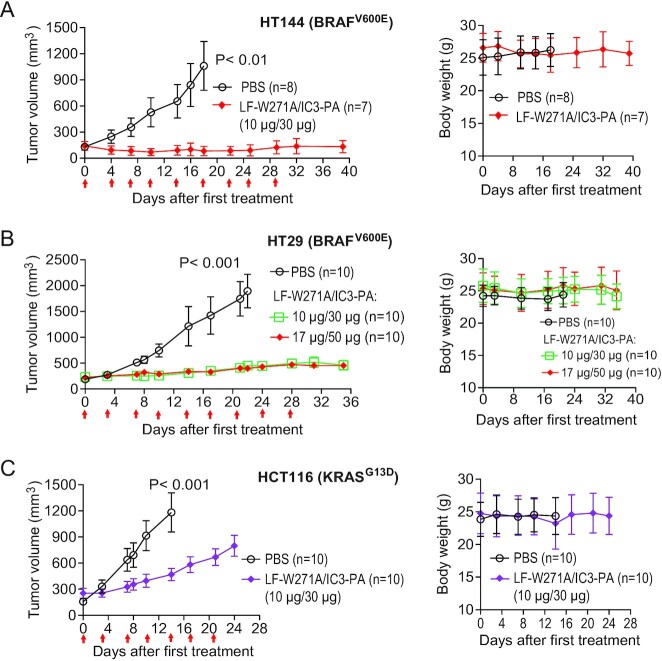
Antitumor activity of our reengineered toxin in long-term experimental cancer therapies. (A)–(C). HT29 (BRAF^V600E^) (A), HT144 (BRAF^V600E^) (B), or HCT116 (KRAS^G13D^) (C) tumor-bearing mice were treated (IP) with PBS or LF-W271A/IC3-PA twice a week as indicated by the red arrows. Tumor weights, mean ± SE. Right panels: Body weights, mean ± SD.

Therefore, next, we sought to evaluate the long-term in vivo efficacy and safety of our tumor-selective MEK inactivator. We implanted human HT-29, HT-144 cells (both with a BRAF^V600E^mutation), or HCT116 cells (with a KRAS^G13D^ mutation) into C57BL/6 J athymic nude (Foxn1^nu/nu^) mice to establish human tumor xenografts. After these tumors were well established (> 200 mm^3^), tumor-bearing mice were treated with PBS or LF-W271A/IC3-PA twice a week as indicated (Figure 5). Notably, our toxin exhibited potent and durable antitumor activity to both HT-29 and HT-144 tumors with the BRAF^V600E^ mutation; while all the PBS-treated mice died or required euthanization within 2 weeks after the first treatment, all the mice treated with the reengineered toxin survived through the 6-week study period with static minimal tumors (Figure 5A and B). Interestingly and surprisingly, the HCT116 (KRAS^G13D^) tumors were also sensitive to the toxin in in vivo, albeit to a lesser extent than the BRAF^V600E^ tumors (Figure 5C). Because HCT116 cells were largely insensitive to the LF-W271A toxin in the ex vivo cytotoxicity assay (Figure 4A; Figure S4A, Supplementary Material), this better in vivo response suggested that targeting the host-derived stromal compartment by the toxin may also contribute to the toxin's antitumor activity. Therefore, our tumor-selective MEK inactivator demonstrates potent in vitro and in vivo antitumor activity, in particular, to tumors with a BRAF^V600E^ mutation. Importantly, our reengineered toxin appears to demonstrate excellent safety features, as we observed no morbidity/mortality or decrease in body weight in any toxin-treated mice during the course of our studies (Figure 5).

### Tumor-selective MEK inactivator exhibits safe and potent antitumor efficacy in immunocompetent mice

**Figure 6.**
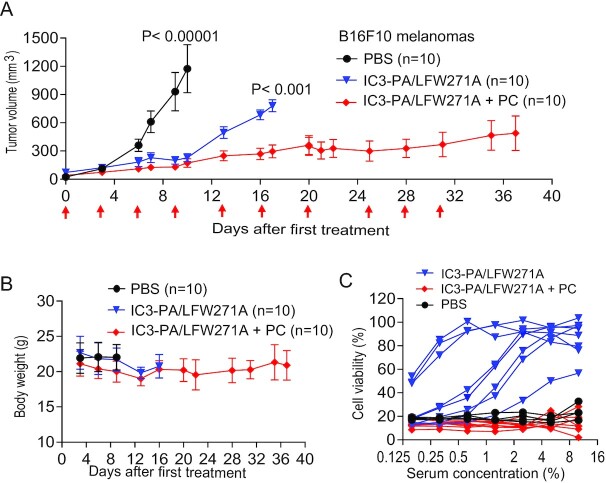
Long-term antitumor activity of LF-W271A/IC3-PA in immunocompetent mice. (A) B16F10 melanoma-bearing immunocompetent C57BL/6 J mice were treated (IP) with PBS, LF-W271A/IC3-PA (10 µg/30 µg), LF-W271A/IC3-PA combined with PC regimen (20 µg pentostatin plus 1 mg cyclophosphamide). PBS vs. all other groups, *P* < 0.00001; LF-W271A/IC3-PA + PC vs. LF-W271A/IC3-PA, *P* < 0.001. Tumor weights, mean ± SE. (B) Body weights of tumor-bearing mice during the course of treatment in (A). Due to tumor burden, the PBS-treated group was euthanized at day 10 after the first treatment. Mean ± SD. (C) Neutralizing activities of antibodies against the toxin. RAW264.7 cells were incubated with PA/LF (100 ng/mL each) for 5 h in the presence of various dilutions of sera obtained from representative mice in (A) (each line represents serum from each mouse) after 2 weeks of therapy (at day 10 for the PBS group). Cell viabilities were determined by an MTT assay as described in Methods. Of note, toxin neutralizing activity could be detected only in sera of the toxin only group.

We further assessed the long-term antitumor activity and safety of our reengineered toxin on B16F10 syngeneic tumors in immunocompetent C57BL/6 J mice. In this immunocompetent setting, long-term therapy with our engineered toxin was expected to induce neutralizing antibodies that block its therapeutic activity. We and others have previously shown that a pentostatin plus cyclophosphamide (PC) regimen, which inhibits B cell proliferation, can efficiently prevent host neutralizing antibody production against therapeutic toxins (16, 38, 39). Therefore, we also coadministered PC along with LF-W271A/IC3-PA, allowing the assessment of long-term therapeutic effects of our reengineered toxin. Strikingly, the combination of our toxin and the PC regimen exhibited a potent, persistent antitumor activity. All toxin/PC-treated mice survived the 6-week study period with only minimum static tumors, whereas all the PBS-treated mice died or were euthanized due to rapid tumor progression (Figures 6A and B). We did observe that the tumors in the toxin alone group were less responsive beyond 10 days after the first treatment. This was likely due to neutralizing antibodies produced in these mice (Figure 6C). Little to no neutralizing antibody activity could be detected from sera obtained from the PC and toxin combination therapy (Figure 6C). Remarkably, no morbidity/mortality or decrease in body weight were observed in mice treated with toxin alone or with the toxin/PC combination.

## Discussion

Small molecule inhibitors have been successfully developed to target BRAF and MEK in cancers with oncogenic mutations in the RAS–RAF–MEK–ERK pathway. Although these inhibitors have helped some patients with metastatic diseases, their utility is limited because they lack tumor specificity and also target normal cells, causing undesirable, even fatal side effects (3, 4). To overcome these limitations, here, we developed a highly tumor-selective MEK inactivator. Generating LF-W271A/IC3-PA required reengineering anthrax toxin's delivery moiety PA and effector protein LF. This tumor targeting toxin exhibits potent and durable antitumor activity with an extremely high therapeutic index (≥ 15). Amazingly, in addition to its compelling antitumor efficacy, our reengineered toxin exhibited excellent safety features. In particular, in this study, we did not observe any morbidity/mortality among > 100 tumor-bearing mice treated with the toxin.

This extremely high tumor specificity is derived from the following properties: IC3-PA needs the simultaneous presence of two distinct tumor-associated proteases, MMPs and uPA, for activation; it also requires binding to the CMG2 receptor which is expressed at high levels on tumor tissues; further, the novel effector moiety LF-W271A can specifically target the ERK pathway while sparing the WT LF's “off-target” proteolytic activity toward p38 and JNK pathways. Intriguingly, while showing much lower in vivo toxicity, LF-W271A has antitumor activity equal to WT LF. This demonstrates that the antitumor activity of LF is mostly due to its inhibitory effect on MEK–ERK signaling, and that inactivation of the p38 and JNK pathways is less relevant for tumor targeting and may cause dose-limiting toxicity.

ERK pathway inhibition by small-molecule MEK inhibitors can release a negative feedback, often leading to pathway reactivation (Figure 4B). Thus, pathway reactivation is a common mechanism through which cancer cells develop resistance to the small-molecule MEK inhibitors. In this regard, since the toxin can irreversibly (proteolytically) inactivate MEK1/2, pathway reactivation is unlikely to occur in cancers treated with our engineered toxin. Therefore, its high tumor specificity and its potent enzymatic activity provide clear advantages of LF-W271A/IC3-PA over the small molecule MEK inhibitors as a potent tumor selective MEK inactivator in tumor targeting.

Cancer cells with BRAF activation mutations are sensitive to small-molecule MEK inhibitors. In line with this, we found that human cancer cells with BRAF^V600E^ are susceptible to LF-W271A toxins, whereas cancer cells with oncogenic KRAS mutations are less sensitive. Therefore, we expected and subsequently demonstrated that tumor xenografts from human cancer cells with BRAF^V600E^ (such as HT29 and HT144) are sensitive to LF-W271A/IC3-PA. However, tumors derived from the cancer cells that are insensitive in vitro to the toxin (such as HCT116 cells) were responsive to in vivo toxin treatment, albeit to a lesser extent than the BRAF^V600E^ tumors. This suggests that targeting the host-derived tumor stromal compartment may also be an important antitumor mechanism of our toxin.

As a “foreign” protein to the host, long-term therapy with our reengineered toxin may induce neutralizing antibodies that may block therapeutic activity. However, recently the field has witnessed rapid progress to solve this immunogenicity issue. A wide range of approaches have been employed including using a B-cell depleting cyclophosphamide/pentostatin (PC) regimen to prevent neutralizing antibody production against bacterial toxin-based immunotoxins in both mice and humans (16, 38, 39). Interestingly, this PC regimen does not appear to affect patients’ antitumor immunity (39). In our study, we found that this PC-based regimen was extremely safe and effective in preventing neutralizing antibody production against our tumor-selective MEK inactivator in mice, allowing repeated use of our toxin in tumor therapy in immunocompetent mice. More recently, Selecta Biosciences has developed tolerogenic nanoparticles comprised of biodegradable polymers that encapsulate rapamycin. These tolerogenic nanoparticles can selectively inhibit antibody production against foreign antigens, allowing long-term therapy in mice (40, 41). This technology is an alternative approach to allow our engineered toxins to be used for long-term antitumor therapy.

Together, our reengineered tumor selective MEK inactivator LF-W271A/IC3-PA has a potent and durable antitumor activity with an extremely high therapeutic index, meriting its further clinical evaluation. Although our approach is particularly effective in treating tumors with the BRAF^V600E^ mutation, it is also active in cancers without oncogenic BRAF mutations via indirectly targeting the host-derived tumor stromal compartment.

## Methods

### Proteins and reagents

Recombinant PA and PA variants, LF and LF variants were purified from supernatants of BH500, an avirulent, sporulation-defective, protease-deficient *B. anthracis* strain, as described previously (42, 43). This expression/purification approach typically produces > 50 mg/L of overnight culture of highly purified lipopolysaccharide-free recombinant protein. PA-I656Q is a CMG2-specific PA variant. PA-L1 is an MMP-activated PA variant, in which the furin-cleavage sequence RKKR (residues 164 to 167) is replaced with a MMP substrate sequence GPLGMLSQ (14). PA-U2 is a urokinase-activated PA variant with furin-cleavage sequence changed to PGSGRSA (13). IC3-PA is a new version of intermolecular complementing PA, consisting of PA-L1-I207R/I656Q and PA-U2-R200A/I656Q, an improved version of the previously described IC2-PA combination (17, 20). FP59 is a fusion protein of LF amino acids 1 to 254 and the catalytic domain of *P. aeruginosa* exotoxin A that kills cells by ADP-ribosylation of eEF2 after delivery to cytosol by PA (44, 45). The features of these recombinant proteins are summarized in Table S1 (Supplementary Material). MTT (3-[4,5-dimethylthiazol-2-yl]-2,5-diphenyltetrazolium bromide) and pentostatin (SML0508) were from Sigma (Atlanta, GA). Cyclophosphamide (NDC10019-957-01) was from Baxter Healthcare (Deerfield, IL). PI3K inhibitor BEZ235 (S1009), MMP inhibitor GM6001 (S7151), UK-371, and 804 HCl (S8457) were from Selleckchem.

### Cells and cytotoxicity assay

All cultured cells were grown at 37°C in a 5% CO_2_ atmosphere. Murine B16F10 melanoma cells and LLC cells (46) were obtained from Dr. Judah Folkman (Harvard Medical School, Boston), and human lung carcinoma A549 cells, colorectal carcinoma Colo205, HT29, HCT116 cells, melanoma HT144, and A2058 cells were from NCI-60 cell set. All tumor cells were cultured in DMEM supplemented with 10% fetal bovine serum.

For cytotoxicity assays, cells grown in 96-well plates (30% confluence) were incubated with various concentrations of PA or LF variant proteins for 48 or 72 h. Cell viabilities were then assayed by MTT as described previously (47), and are expressed as % of MTT signals of untreated cells. In Schild plot analyses, the cells were incubated with various concentrations of PA plus FP59 (constant at 1.9 nM) for 1.5 h in the presence of different fixed concentrations of a nontoxic PA competitor, PA-U2(D512K) or PA-U2(D512K)-I656Q. Then the toxins were removed, and the cells were cultured 48 h for cell viability analyses.

To generate LLC and B16F10 CMG2-KO cells for by CRISPR gene editing, we cloned the mouse CMG2 sgRNA sequence (ACCATCTTATGCAGAGAACG) targeting CMG2’s extracellular domain into the pSpCas9-2A-puro vector (Addgene, #48139). Cloning of CMG2 sgRNA into pSpCas9-2A-Puro was done by following the protocol described by Feng Zhang's laboratory (48). X-tremeGENE 9 DNA Transfection Reagent was used for transfection of the plasmids into the indicated cells following the manufacturer's manual (Roche, Cat. #06366236001). We transfected the resulting CMG2 sgRNA construct into LLC or B16F10 cells, resulting in the respective CMG2-KO cells.

For assessing the effects of LF or LF-W271A on the ERK, p38, and JNK pathways, the cells were incubated with various concentrations of LF/PA or LF-W271A/PA for 2 h. Then cell lysates were prepared in the modified RIPA lysis buffer containing protease inhibitors as described (47). Cell lysates were separated on SDS-PAGE gels, transferred onto nitrocellulose membranes, and analyzed by western blotting using anti-MEK1 (#07–641, Upstate Technology), -MEK2 (#67410, Proteintech), -MEK3 (#8535, Cell Signaling), -MEK4 (#67333, Proteintech), -MEK6 (#8550, Cell Signaling), MEK-7 (#4172, Cell Signaling), anti-P-ERK (#4695, Cell Signaling), -P-p38 (#4511, Cell Signaling), or anti-P-JNK (#9255, Cell Signaling) antibody.

To analyze BRAF and RAS mutation status in B16F10 cells, total RNA was prepared from the cells using TRIzol reagent (Invitrogen, Carlsbad, CA), and was used to synthesize single-strand cDNA using the SuperScript IV First-Strand Synthesis System following the manufacturer's manual (Invitrogen, cat. no. 18091050). Full-length BRAF, KRAS, NRAS, and HRAS cDNA fragments were amplified by reverse transcriptase-PCR and sequenced, revealing that B16F10 cells contain WT BRAF, KRAS, NRAS, and HRAS genes.

### Mice and tumor studies

For tumor studies, 10- to 14-week-old male and female mice were used. C57BL/6 J mice and C57BL/6 J athymic nude (*Foxn1^nu/nu^*) mice were obtained from the Jackson Laboratory (Bar Harbor, Maine). To grow syngeneic tumors, 5 × 10^5^ cells/mouse B16F10 or B16(CMG2-KO) melanoma cells or LLC lung carcinoma cells were injected in the midscapular subcutis of the preshaved C57BL/6 J mice (49). Visible B16 tumors and LLC tumors (about 50 mm^3^) usually formed 6 to 8 days after inoculation. For human tumor xenografts, 5 × 10^6^ cells/human HT29 colorectal carcinoma cells, HCT116 cells, or HT144 melanoma cells were injected intradermally into C57BL/6 J athymic nude (*Foxn1^nu/nu^*) mice. Visible HT29, HCT116, and HT144 tumors usually formed 10 to 12 days after inoculation. Tumors were treated when they became visible or at later stages and measured with digital calipers (FV Fowler Company, Inc., Newton, MA). Tumor volumes were estimated with the length, width, and height tumor dimensions using formulas: tumor volume (mm^3^) = ½ (length in mm × width in mm × height in mm). Tumor-bearing mice were randomized into groups and injected intraperitoneally following schedules indicated in the figures. Mice were weighed and the tumors measured before each injection. Mice were euthanized when tumors reached 2.0 cm in diameter or showed signs for pathological stresses, such as weight loss of up to 20% of the total body weight, decreased food and water intake, dehydration, weakness, or difficulty in breathing.

### Measurement of toxin-neutralizing antibodies

B16F10 tumor-bearing mice from various treatment groups were terminally bled and sera prepared. To titrate toxin neutralizing activity in the sera, RAW264.7 cells, which are quickly killed by anthrax lethal toxin via pyroptotic cell death, were incubated with 125 ng/mL PA plus 125 ng/mL LF (amounts that kill > 95% of the cells) in the presence of various dilutions of the sera for 5 h, followed by an MTT assay to determine cell viability.

### Statistical analysis

Statistical differences in tumor size were calculated using the two-tailed Student's t test when two treatment groups are compared or a one-way ANOVA when comparing more than two groups. Survival curves were compared using a two-tailed Log-rank test using GraphPad Prism. *P* < 0.05 was considered as a significant difference.

## Supplementary Material

pgac104_Supplemental_File

## Data Availability

All data is included in the manuscript and/or supporting information.
