# Supplementary material for: A potent tumor-selective ERK pathway inactivator with high therapeutic index
Source: PNAS Nexus. 2022 Jul 1;1(3):pgac104. doi: 10.1093/pnasnexus/pgac104 (PMC9308561; doi:10.1093/pnasnexus/pgac104)
Supplement: pgac104_Supplemental_File [file pgac104_supplemental_file.docx]

**Figure S1**. PA-I656Q is one of the PA variants that selectively use the CMG2 receptor rather than TEM8 for cytotoxicity

**A**. CMG2-selective PA variants. TEM8-expressing cells and CMG2-expressing cells are CHO mutant cells only express CMG2 or TEM8 receptor, respectively. These cells were incubated with various concentrations of PA variants plus FP59 (100 ng/mL) for 48 h, and the cell viability was assessed by an MTT assay.

**B**. Apparent binding affinity of PA-I656Q to the CMG2-expressing and TEM8-expressing CHO cells. The cells were incubated with various concentrations of PA plus FP59 (1.9 nM) and different concentrations of non-toxic binding competitor PA-U2(D521K)-I656Q (equivalent to PA-I656Q in receptor binding) as indicated for 1.5 h. Then the toxin proteins were removed and the cells were incubated with fresh medium until 48 h when the cell viability was determined. In Schild plot analysis, the EC_50_ values (left panels) of PA determined in the presence of various fixed concentrations of PA-U2(D521K)-I656Q were used to fit the equation Y= -Log(X+10Log K_d_)-P (where Y= -Log(EC_50_) (nM), X=[PA-U2(D521K)-I656Q] (nM), P is a constant) to determine K_d_s of PA-U2(D521K)-I656Q (equivalent to PA-I656Q) to the respective receptor-expressing cells. The right panels are the regression curves obtained using Prism program. PA proteins: 83 ng/mL = 1 nM; FP59: 100 ng/mL = 1.9 nM.

**C**. Apparent binding affinity of PA to the CMG2-expressing and TEM8-expressing CHO cells. Analyses were performed as in (B), but competitor PA-U2(D521K) (equivalent to wildtype PA in receptor binding) was used instead.


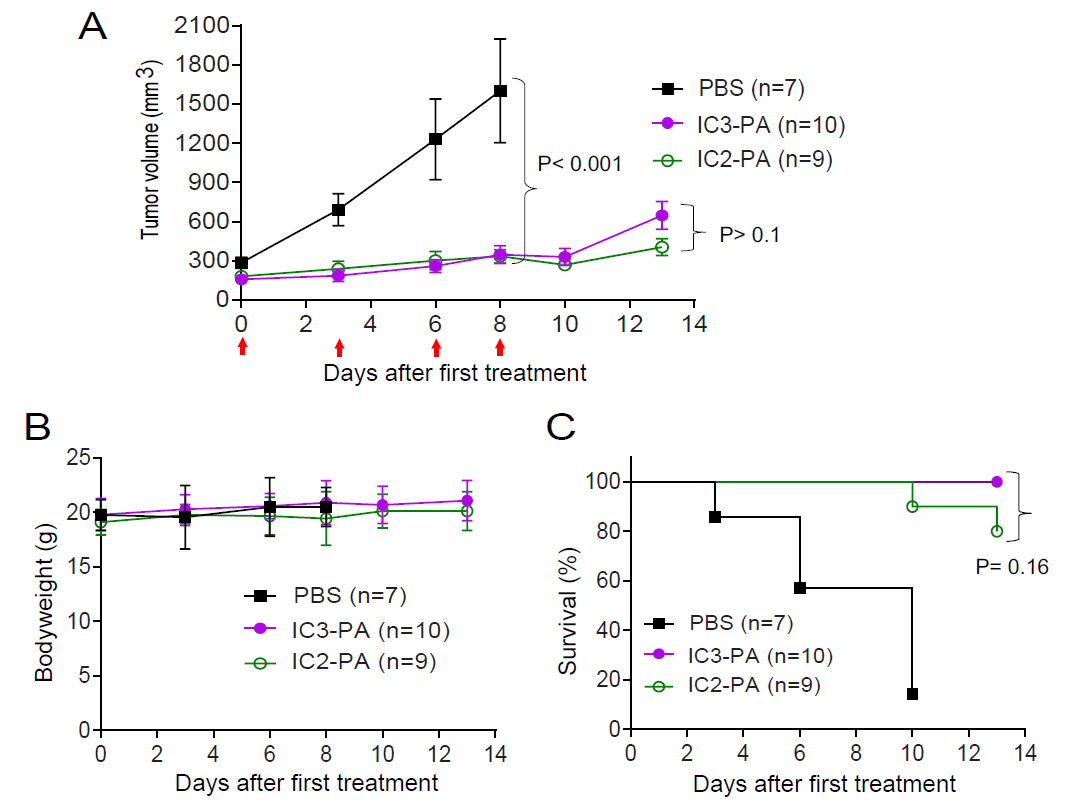
**Figure S2**. IC3-PA exhibits similar antitumor activity as IC2-PA

**A-C**. B16F10 tumor-bearing mice were treated (I.P.) with PBS, LF/IC3-PA (6.7 µg/20 µg), or LF/IC2-PA (6.7 µg/20 µg) as indicated by the red arrows (A). Bodyweight (B) and survival (C) of each group were monitored. Tumor weights, mean ± SE. Bodyweights, mean ± SD. Of note, the PBS group was terminated earlier due to reaching euthanasia criteria (tumors > 2 cm in diameter or ulceration).

**Figure S3**. Differential proteolytic activity of LF variants to MEK2 vs. MEK3

B16 cells were incubated with indicated dilutions of the LF variant-containing supernatants from the expressing host BH500 strains for 3 h in the presence of 100 ng/mL PA. Cell lysates were then prepared, followed by Western blotting using anti-MEK2 and -MEK3 antibodies. Upper panels, Western blotting against MEK3. Lower panel, the blots in upper panels were re-probed with an anti-MEK2 antibody. Of note, the MEK3 bands were still visible below the MEK2 bands in the lower panels. LF-W271A (boxed lanes) was selected for further analyses.

**Figure S4**. Human cancer cells with oncogenic BRAF^V600E^ mutation are more sensitive to the MEK inhibition by LF-W271A than the cells with KRAS mutations

A. Cancer cells with the BRAF^V600E^ or KRAS mutation were incubated with various concentrations of LF-W271A in the presence of 500 ng/mL PA for 72 h, followed by an MTT assay for assessing cell viability. Means ± SD.

B. Cells were treated with concentrations of PA in the presence of 100 ng/mL FP59 for 72 h, followed by an MTT assay for assessing cell viability. Means ± SD.

C. HT29 cells were incubated with 500 ng/mL LF-W271A/PA or LF/PA for 3 h. The toxins were then removed. The cell lysates were prepared at indicated time points after toxin removal, followed by Western blotting using anti-Phospho-ERK (T202/Y204) antibody.

**Table S1**. Properties of anthrax toxin-based tumor-selective MEK inactivators

| **Delivery component** | **Activated by** | **Receptor specificity** |
| --- | --- | --- |
| PA (wildtype) | Furin | CMG2, TEM8 |
| PA-L1 | MMPs | CMG2, TEM8 |
| PA-U2 | uPA | CMG2, TEM8 |
| IC2-PA  = PA-L1-I207R + PA-U2-R200A | MMPs, uPA | CMG2, TEM8 |
| PA-L1-I656Q | MMPs | CMG2 |
| PA-U2-I656Q | uPA | CMG2 |
| **IC3-PA**  = PA-L1-I207R/I656Q + PA-U2-R200A/I656Q | MMPs, uPA | CMG2 |
|  | | |
| **Effector component** | **Substrate** | |
| LF (wildtype) | MEK1, MEK2, MEK3, MEK4, MEK6 | |
| **LF-W271A** | MEK1, MEK2 | |

**Table S2**. Mutations in human cancer cell lines used in this study

| **Human Cancer cell line (ATCC)** | **Oncogenes** | **Tumor suppressor genes** |
| --- | --- | --- |
| SW620  (Colorectal adenocarcinoma) | KRAS p.G12V | APC p.Q1338* (nonsense)  TP53 p.R273H  TP53 p.P309S |
| MDA-MB-231  (Mammary adenocarcinoma) | KRAS p.G13D  BRAF p.G464V | TP53 p.R280K |
| A549  (Lung carcinoma) | KRAS p.G12S | CDKN2A p.0? |
| HCT116  (Colorectal carcinoma) | KRAS p.G13D  PIK3CA p.H1047R | CDKN2A p.R24fs*20 |
| AsPc-1  (Pancreatic adenocarcinoma) | KRAS p.G12D | TP53 p.C135fs*35  CDKN2A p.L78fs*41 |
|  |  |  |
| RKO  (Colon carcinoma) | BRAF p.V600E  PIK3CA p.H1047R | unknown |
| A2058  (Melanoma) | BRAF p.V600E | TP53 p.V274F  PTEN p.L112Q  PTEN p.V175fs*3 |
| Colo205  (Colorectal adenocarcinoma) | BRAF p.V600E | APC p.T1556fs*3  TP53 p.Y103_L111>L |
| HT29  (Colorectal adenocarcinoma) | BRAF p.V600E  PIK3CA p.P449T | APC p.T1556fs*3  TP53 p.R273H  SMAD4 pQ311* |
| HT144  (Melanoma) | BRAF p.V600E | CDKN2A p.0?  PTEN p.? |

Data was extracted from ATCC cell lines by gene mutation database (<https://www.academia.edu/43984296/ATCC_CELL_LINES_BY_GENE_MUTATION>)
